# Ectopic ACTH Syndrome Emerging 5 Years after the Diagnosis of Neuroendocrine Tumor

**DOI:** 10.1155/2019/6583467

**Published:** 2019-05-28

**Authors:** Minghao Liu, Diane Hamele-Bena, John Ausiello, Gabrielle Page-Wilson

**Affiliations:** ^1^Endocrinology Division, Department of Medicine, Columbia University Medical Center, 650 West 168th St., Room 2012, New York, NY 10032, USA; ^2^Department of Pathology and Cell Biology, Columbia University Medical Center, Physicians and Surgeons Building, Room 404, 630 W 168th St., New York, NY 10032, USA; ^3^Endocrinology Division, Department of Medicine, Columbia University Medical Center, Harkness Pavilion, 9th floor, 180 Fort Washington Ave., New York, NY 10032, USA

## Abstract

Ectopic ACTH syndrome (EAS) arising years after the diagnosis of a neuroendocrine tumor (NET) is exceedingly rare. We describe a case of EAS occurring five years after the diagnosis of a metastatic lung NET in a 61-year-old woman. She presented with severe hypokalemia but was not overtly Cushingoid on exam. Serum cortisol was 61mcg/dL after an overnight 1mg dexamethasone suppression test (<1.8mcg/dL) and urinary free cortisol was 7544 mcg/24h (<45mcg/24h), establishing the diagnosis of Cushing's syndrome. Plasma levels of peptides which have been associated with EAS, Agouti-related peptide (AgRP) and the ACTH precursors POMC (31-kDa) and pro-ACTH (22-kDa), were elevated. Metyrapone was initiated, but hypercortisolism persisted and the patient succumbed to pneumonia shortly after presentation. Retrospective examination of biopsy tissues showed rare ACTH immunoreactivity at the time of initial diagnosis, followed by staining in a greater proportion of cells as the disease progressed, consistent with EAS arising years after the diagnosis of NET. Given the increase in mortality associated with EAS, this unusual case highlights the importance of early detection and raises the possibility that early immunohistochemical stains for ACTH and measurements of ACTH precursors may facilitate the identification of NETs at high risk for EAS.

## 1. Introduction

Neuroendocrine tumors (NETs) arise from cells that have the ability to synthesize and secrete various peptides and amines, some of which may be metabolically active and give rise to distinct paraneoplastic syndromes. Lung NETs have an annual incidence of 0.2-2.0 per 100,000 and 1% of these cases are complicated by ectopic ACTH syndrome (EAS), a rare condition characterized by tumoral ACTH production, hypercortisolism, and increased mortality [1, 2]. While EAS is classically associated with small-cell lung carcinomas and carcinoids, it can be a paraneoplastic complication of neuroendocrine carcinoma (NEC), an aggressive form of NET [3]. We describe a rare case of a lung neuroendocrine carcinoma complicated by EAS five years after the tumor diagnosis. Tumoral ACTH immunoreactivity was retrospectively noted on the initial diagnostic biopsy specimen and plasma POMC concentrations were elevated at onset of EAS.

## 2. Case Presentation

A 61-year-old woman with Hashimoto's thyroiditis was hospitalized for new-onset hypokalemia two weeks following initiation of hydrochlorothiazide. Five years earlier, the patient had been diagnosed with a metastatic lung neuroendocrine tumor, while imaging for chronic cough revealed a lung lesion, subcarinal lymph node, and liver nodule. Follow-up PET scan had shown FDG avidity at those sites without brain involvement. Subcarinal node biopsy showed malignant cells with neuroendocrine features including nuclear molding, “salt and pepper” chromatin, and apoptosis. Immunohistochemical staining was positive for chromogranin, synaptophysin, and CD56, with an initial Ki67 index <5%. Plasma chromogranin A was 120 ng/mL (0-95 ng/mL). Morning cortisol and ACTH were normal at 9.3 mcg/dL (5.0-25.0 mcg/dL) and 19 pg/mL (6-58 pg/mL), respectively. Treatment with temozolomide and capecitabine was initiated with near resolution of the liver metastasis and stable disease was achieved for 2 years. However, disease progression characterized by new dedifferentiated metastases (Ki-67 index > 20%) to the liver, vertebra, brain (parietal region), shoulder soft tissue, ovary, and orbits subsequently occurred. Sequential treatment with radioembolization of hepatic metastases, octreotide, gamma knife, everolimus, and lanreotide was pursued over the course of 3 years.

On the day of admission, the patient presented from home with diffuse pain, chronic fatigue, and weakness. She denied fevers, chills, weight changes, and easy bruisability and had no history of recent fractures. Exam revealed an ill-appearing woman with a BMI of 27.8 and normal vital signs. She was not overtly Cushingoid; there was no facial plethora, supraclavicular/dorsocervical fullness, acanthosis, hirsutism, striae, or ecchymoses. Proximal muscle weakness, trace pretibial edema, and 1+ reflexes without delayed relaxation were noted. Laboratory evaluation demonstrated hypokalemia, metabolic alkalosis, mild hypernatremia, elevated hemoglobin A1c, transaminitis, elevated alkaline phosphatase, and hypothyroidism (Table 1). Morning cortisol was 48 mcg/dL (4.8-19.5) and was 61 mcg/dL after a 1 mg dexamethasone suppression test (<1.8). ACTH was 141 pg/mL (6-58) and urinary free cortisol was 7544 mcg/24h (<45). MRI showed multiple new orbital and brain metastasis, including a new 0.97 x 0.74 cm sellar/suprasellar lesion involving the pituitary stalk. The patient was found to have diabetes insipidus and treated with nightly desmopressin.

Although pituitary Cushing's was a possible diagnosis when considering a sellar/suprasellar lesion and a high plasma ACTH level in isolation, the patient had several features that pointed away from Cushing's disease and towards EAS: she had rapid onset of symptoms, severe hypokalemia, and an extremely high urinary free cortisol level (7544 mcg/24h). Furthermore, the appearance of the sellar/suprasellar lesion late in the timeline after spread of the lung NET to other regions of the body, the lesion's involvement of the pituitary stalk, and the patient's diabetes insipidus all strongly suggested that it was a metastasis of the NET. We did not perform inferior petrosal sinus sampling because of low clinical suspicion for Cushing's disease and because the patient was too ill to undergo this procedure.

Given high suspicion for EAS, plasma POMC and AgRP were measured as part of a Columbia University Medical Center IRB-approved research protocol in patients with ACTH-dependent Cushing's syndrome [4]. POMC was measured using an immunoradiometric assay (IRMA) that detects POMC (31-kDa) and pro-ACTH (22-kDa) but not ACTH or other ACTH precursors, as previously described [5], and AgRP was measured by ELISA [4]. POMC level was 80 fmol/mL (7-32 fmol/mL) and AgRP level was 884 pg/mL (42-118 pg/mL), supporting the diagnosis of EAS [4].

Hydrochlorothiazide was discontinued but hypokalemia requiring 120-200 mEq of daily potassium chloride repletion persisted until spironolactone was uptitrated to 50mg twice daily. Home levothyroxine was increased from 50 to 75mcg daily. Ketoconazole was deferred in the setting of diffuse hepatic metastases. Metyrapone was initiated and aggressively uptitrated, but hypercortisolism persisted and the patient died from multifocal pneumonia shortly after presentation.

**Figure 1:**
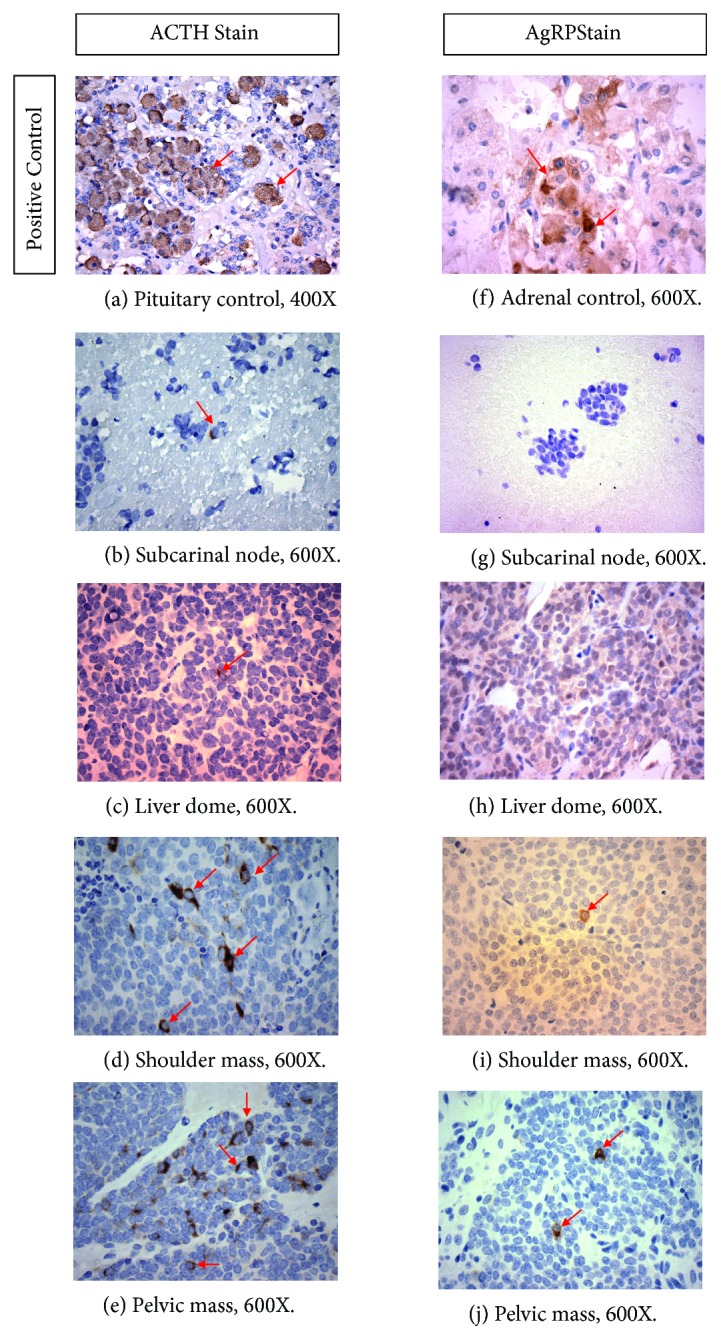
*Histopathology and immunohistochemistry of metastatic NEC biopsy specimens*: (a) Positive control: ACTH staining in normal pituitary tissue. Stained cells (red arrows) have brown cytoplasm. (b) Rare ACTH-positive cells in the subcarinal node that provided the initial diagnosis of NET and in (c) liver dome biopsied 3.5 years later. Increase in staining intensity and number of cells staining positive for ACTH in (d) shoulder mass and (e) pelvic mass, biopsied 4 and 4.5 years after initial diagnosis, respectively. (f) Positive control: AgRP staining in normal adrenal tissue. No staining for AgRP in specimens biopsied earlier in the disease course: (g) subcarinal node and (h) liver dome. Specimens biopsied later: (i) shoulder mass and (j) pelvic mass: show rare cells staining for AgRP.

Retrospective histologic examination of the subcarinal lymph node and liver biopsy specimens from the time of initial NET diagnosis confirmed ACTH immunoreactivity (Ventana Medical Systems: AB2335961) in rare malignant cells (Figure 1). A progressive increase in the number of malignant cells displaying strong ACTH immunoreactivity was observed in subsequent biopsy specimens obtained at least 3.5 years after diagnosis. While AgRP immunoreactivity (R&D Systems: AB355537) was absent from the initial tumor specimens, rare cells with AgRP immunoreactivity were observed in later biopsy specimens.

## 3. Discussion

This case demonstrates the rare occurrence of ectopic ACTH syndrome five years after the diagnosis of a lung neuroendocrine tumor. While EAS is known to be a potential paraneoplastic complication of a diverse array of NETs, several features of this case are unique. EAS is usually associated with typical carcinoids or small cell lung cancers [1]. While it has been observed with other tumors including pheochromocytomas, pancreatic islet-cell tumors, medullary thyroid carcinomas, and prostate carcinomas, its occurrence in the setting of non-small-cell lung cancer is exceedingly rare [6]. There are case reports of EAS complicating large cell neuroendocrine carcinoma of the lung [7] and lung adenocarcinoma. However, the primary malignancy in this case was an aggressive lung neuroendocrine carcinoma with neither small cell, large cell, nor glandular cell features. The tumor was initially diagnosed as a neuroendocrine tumor with a low proliferative index. However, three and a half years into her course, the tumor exhibited more aggressive characteristics including a high mitotic count and an elevated Ki-67 index. Given that the proliferative index is scored on only a small portion of the tumor specimen, it is possible that the original pathologic assessment was inadequate. Alternatively, the original NET may have progressed over time to a high-grade neuroendocrine carcinoma.

The second noteworthy feature of this case is the markedly delayed occurrence of EAS. The temporal onset of EAS in patients with neuroendocrine tumors is categorized as synchronous, diagnosed within the 3-month period before or after tumor diagnosis; metachronous, diagnosed greater than 3 months after tumor diagnosis; or cyclic [1]. In the majority of cases, EAS occurs synchronously and it is not uncommon for patients to present with paraneoplastic EAS before a cancer diagnosis is made [8]. There is little available data about the temporal onset of EAS specifically in patients with lung neuroendocrine tumors. However, in a large cohort of 918 thoracic and gastroenteropancreatic NETs evaluated by Kamp et al., which included 51 lung/bronchial NETs, paraneoplastic Cushing's was identified metachronously in only 4 of the 29 cases associated with EAS [1]. The duration between the onset of hypercortisolism and the initial NET diagnoses was not reported. In our extensive review of the literature, we identified only two reported cases of metachronous EAS associated with lung NET. In the first case, EAS was diagnosed 12 years after the initial diagnosis of a malignant bronchial carcinoid and heralded recurrent disease [9]. In the second case, the delayed occurrence of EAS was documented 3 years after the diagnosis of malignant carcinoid or small-cell carcinoma of the lung in the setting of new brain metastases [10]. The occurrence of paraneoplastic EAS is most prevalent among NET patients with advanced (stage IV) disease. This clinical feature can complicate the diagnosis because many of the pathognomonic features of hypercortisolism may be obscured by cancer-related symptoms and chemotherapy-associated side effects.

Regular screening for Cushing's may have improved our patient's outcome, but given the rarity of EAS and the potential for its delayed occurrence, routine surveillance for EAS in all NETs is not likely to be cost effective. The ability to preemptively identify NET patients at high risk for EAS may facilitate the timely diagnosis and treatment of this morbid paraneoplastic complication. In this case report, the plasma cortisol and ACTH levels were normal when the initial NET diagnosis was made. However, the diagnostic tumor specimen was immunoreactive for ACTH, which may have heralded the tumor's potential for ACTH synthesis. Over time, there was an increase in tumoral ACTH staining followed by the eventual onset of EAS, which suggests that, while rare, it is possible for NETs to transform from clinically silent to hormonally functional tumors even years after their diagnosis. The mechanisms underlying this transformation may parallel those observed when silent corticotroph adenomas transform into functioning corticotroph adenomas, in part by increasing levels of the processing enzymes that mediate the conversion of POMC to ACTH [11]. While it is also possible that the development of Cushing's syndrome reflects an overall increase in the burden of ACTH immunoreactive tumor, a lack of concordance between tumor size and ACTH levels has been previously observed in EAS [3].

To date, specific biochemical markers indicative of NET's potential for clinically relevant ACTH secretion have not been comprehensively validated. However, elevations in POMC, the peptide precursor to ACTH, have been associated with occult and overt EAS [4]. Most nonpituitary tissues transcribe a short POMC mRNA that is not translated, but unmethylation of an upstream promoter allows ectopic ACTH-producing tumors to transcribe a longer mRNA that is translated into POMC protein [12]. However, in EAS, tumoral dedifferentiation is often associated with aberrant posttranslational processing of POMC, resulting in high circulating concentrations of unprocessed POMC peptide and ACTH precursors [13]. Consistent with these observations, we confirmed elevated plasma POMC (31-kDa) and pro-ACTH (22-kDa) concentrations in this case.

In addition, we observed a markedly elevated AgRP level in this patient. AgRP is most well known as a hypothalamic neuropeptide that stimulates appetite and inhibits energy expenditure by inhibiting the effect of alpha-MSH at melanocortin receptors. Plasma AgRP has been shown to reflect hypothalamic AgRP; it rises with fasting, falls with refeeding, and is inversely associated with BMI [14–17]. While AgRP is regulated by leptin, it is also regulated by cortisol in both animals and humans. Cortisol stimulates AgRP, which may partially explain why the AgRP is high in this case despite the patient having a BMI that is not low [17]. Another likely explanation for the elevated AgRP is the production of AgRP by the metastatic lung NET. AgRP is known to be expressed in tissues other than the hypothalamus including the lungs, adrenals, kidneys, and gonads [18, 19]. Furthermore, elevated plasma levels of AgRP have been observed in EAS and such elevations may be associated specifically with NETs arising from the lungs and the adrenals [4, 18]. Our patient's metastatic lung NEC was focally immunoreactive for AgRP, providing strong support for the tumoral origin of this circulating peptide. Studies establishing concordance between elevations in plasma AgRP and NET AgRP immunoreactivity are needed to support the clinical use of AgRP as a biomarker for EAS. However, the elevations in plasma POMC and AgRP in this case highlight the potential utility of these neuropeptides as tumor markers.

Delayed EAS occurring years after a cancer diagnosis is rare. However, it is imperative for providers to be aware of this potential complication because timely diagnosis and treatment can improve survival. The presence of ACTH immunoreactivity in diagnostic NET biopsy specimens may facilitate the early identification of patients at the highest risk of developing EAS. The presence of elevations in circulating neuroendocrine peptides, particularly POMC, may also serve as a specific marker of a NET's potential for ectopic ACTH secretion.

## Figures and Tables

**Table 1:** Laboratory values on admission.

Laboratory Test	Patient's Value	Reference Range
Sodium (mmol/L)	147	137-145
Potassium (mmol/L)	2.6	3.5-5.1
Bicarbonate (mmol/L)	37	19-27
Creatinine (mg/dL)	0.67	0.50-0.95
AST (units/L)	46	10-37
ALT (units/L)	78	9-50
Alkaline Phosphatase (units/L)	260	40-129
Hemoglobin A1c (%)	6.0%	<5.7%
TSH (mIU/L)	0.22	0.41-4.81
Free T4 (ng/dL)	0.63	0.83-1.90
Total T3 (ng/dL)	56.4	78-158

## Additional Points

(i) Polyclonal Adrenocorticotropin Antibody, Ventana Medical Systems Cat# 760-2708, RRID:AB_2335961, http://antibodyregistry.org/search.php?q=AB_2335961. (ii) Human AgRP Affinity Purified Polyclonal Antibody, R and D Systems Cat# AF704, RRID:AB 355537, http://antibodyregistry.org/search.php?q=AB_355537. (iii) POMC levels were assessed using an immunoradiometric assay (IRMA) based on two monoclonal antibodies: the capture antibody is directed towards ACTH and the detection antibody is directed against *γ*MSH. Thus, this IRMA detects the only two peptides containing both epitopes: POMC (31-kDa) and pro-ACTH (22-kDa). It does not detect other ACTH precursors. The antibodies for the IRMA are obtained from Dr. Anne White's lab (University of Manchester, Salford, UK) and the assay is performed as her group previously described [5].
